# Objective Endoscopic Analysis with Linked Color Imaging regarding Gastric Mucosal Atrophy: A Pilot Study

**DOI:** 10.1155/2017/5054237

**Published:** 2017-11-15

**Authors:** Kazuhiro Mizukami, Ryo Ogawa, Kazuhisa Okamoto, Mitsutaka Shuto, Kensuke Fukuda, Akira Sonoda, Osamu Matsunari, Yuka Hirashita, Tadayoshi Okimoto, Masaaki Kodama, Kazunari Murakami

**Affiliations:** Department of Gastroenterology, Oita University, 1-1 Idaigaoka, Hasama, Yufu, Oita 879-5593, Japan

## Abstract

**Objectives:**

We aimed to determine whether linked color imaging (LCI), a new image-enhanced endoscopy that enhances subtle differences in mucosal colors, can distinguish the border of endoscopic mucosal atrophy.

**Methods:**

This study included 30 patients with atrophic gastritis. In endoscopy, we continuously took images in the same composition with both LCI and white light imaging (WLI). In each image, the color values of atrophic and nonatrophic mucosae were quantified using the International Commission on Illumination 1976 (L^∗^, a^∗^, b^∗^) color space. Color differences at the atrophic border, defined as Euclidean distances of color values between the atrophic and nonatrophic mucosae, were compared between WLI and LCI for the overall cohort and separately for patients with *Helicobacter pylori* infection status.

**Results:**

We found that the color difference became significantly higher with LCI than with WLI in the overall samples of 90 points in 30 patients. LCI was 14.79 ± 6.68, and WLI was 11.06 ± 5.44 (*P* < 0.00001). LCI was also more effective in both of the *Helicobacter pylori*-infected group (*P* = 0.00003) and the *Helicobacter pylori*-eradicated group (*P* = 0.00002).

**Conclusions:**

LCI allows clear endoscopic visualization of the atrophic border under various conditions of gastritis, regardless of *Helicobacter pylori* infection status.

## 1. Introduction

Since the discovery of *Helicobacter pylori* (*H. pylori*) [1], chronic gastritis has attracted attention not only for its symptoms but also as a precursor lesion for gastric cancer. At present, the endoscopic diagnosis of chronic gastritis is one of the most important findings with careful consideration.

Typical endoscopic findings of *H. pylori*-infected gastric mucosa include atrophy, diffuse redness, hyperplastic polyps, gastric xanthoma, intestinal metaplasia, enlarged folds, and nodularity [2–6]. Among these findings, atrophy [7, 8], nodular gastritis [9], and enlarged folds [4] are reported to be associated with gastric cancer. Particularly, the progression of atrophy has been noted to correlate with the risk of developing gastric cancer. Masuyama et al. reported that cancer incidence was higher among patients with advanced gastric mucosal atrophy, and most patients with synchronous or metachronous cancer presented with particularly severe atrophy [10]. Thus, accurate evaluation of the degree of atrophy is expected to enable prediction of not only current but also future development of cancer.

Endoscopic mucosal atrophy is diagnosed based on findings such as loss of folds, discoloration, and enhanced visibility of the vascular pattern, while the progression of mucosal atrophy is classified according to the extent of mucosal involvement [11]. When the border of the lesion is clearly distinguishable in terms of color (e.g., atrophic and nonatrophic mucosae have a different color), it is easy to determine the extent of atrophy. However, when the border is ambiguous in color, it may be difficult to detect atrophy or determine its extent.

Linked color imaging (LCI) (Fujifilm Co., Tokyo, Japan) is a recently developed image-enhanced endoscopy system that emphasizes differences in mucosal color by irradiating with abundant narrow-band short-wavelength light. Therefore, subtle changes in mucosal color are more clear on LCI than on white light imaging (WLI). Although LCI has been reported to facilitate detection of flat lesions indicating early gastric cancer or laterally spreading tumors in the colon [12, 13], the capabilities of LCI for comprehensive findings of gastrointestinal tract such as atrophic gastritis have not been investigated.

In the present study, we used an LCI system to observe and assess atrophic lesions of the gastric mucosa in patients with various *H. pylori* infection status, such as active or eradicated. We subsequently analyzed objective measurements of color differences to determine whether LCI was more helpful than WLI for diagnosing endoscopic atrophy.

## 2. Methods

### 2.1. Instrument

**Figure 1:**
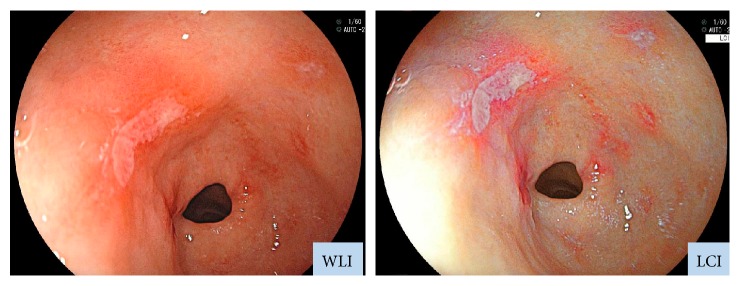
Endoscopic images obtained with WLI and LCI. LCI was developed to enhance colors similar to those of the mucosa. Whereas WLI indicated only erosion, LCI revealed also inflammation, which appeared as redness around the erosion site. Inflammation could be detected on LCI because it appeared redder, while the background mucosa appeared whiter on LCI than on WLI.

We employed an EG-L590ZW endoscope with the LASEREO system (Fujifilm Co., Tokyo, Japan), which is capable of producing light suitable for WLI and LCI. The LCI mode enhances minute color differences in the red spectrum, which is where mucosal colors typically lie. While LCI is based on images captured under light conditions similar to those employed in blue laser imaging-bright mode, subsequent image processing is applied to enhance red (e.g., slightly reddish colors become much redder) and white (e.g., slightly whitish colors become much whiter) (Figure 1) [14].

### 2.2. Study Design

This study targeted patients aged ≥20 years who underwent esophagogastroduodenography (EGD) at Oita University Hospital (Yufu, Oita Prefecture, Japan). The disease cases causing extensive changes in the gastric mucosa, such as liver cirrhosis, were excluded. During EGD, the atrophic border was identified. Following identical compositions, images were continuously captured via WLI and LCI. In each patient, information regarding *H. pylori* infection status and progression of endoscopic atrophy was collected and analyzed. This study was conducted according to the principles of the Declaration of Helsinki and with the approval of the ethics committee of Oita University.

*H. pylori* infection status was established based on biopsies collected during EGD and sampled from two sites, namely from the greater curvature side of antrum and from the greater curvature side of the upper corpus. The biopsy specimens for histopathology were fixed in 10% neutral formalin for 24 hours and subsequently embedded in paraffin. Sections were obtained and underwent hematoxylin and eosin staining and May-Giemsa staining. Active *H. pylori* infection was established in patients found positive on any one of the following tests, rapid urease test, culture, and histopathology, and these patients were included in the *H. pylori*-infected group. Those who were found negative on all tests, presented with endoscopic atrophy, and had a clear history of *H. pylori* eradication were included in the *H. pylori*-eradicated group.

Endoscopic findings of gastric mucosal atrophy were assessed according to the Kimura-Takemoto classification [11], which defines atrophic patterns based on the following characteristic features: C1, when atrophic mucosa is only found in the antrum; C2, when atrophic mucosa is found at the gastric angle or in the lower corpus; C3, when atrophic mucosa is also found in the upper corpus; O1, when atrophic mucosa surrounds the gastric cardia but the folds of the great curvature are relatively maintained; O3, when the entire gastric mucosa is atrophic, and the folds of the greater curvature as a whole disappeared; and O2, representing an intermediate condition between O1 and O3. All endoscopic findings were assessed by experienced endoscopists.

### 2.3. Measurement of Colors

Color differences between atrophic and nonatrophic mucosal areas were measured on WLI- and LCI-acquired images. The color differences at the atrophic border were compared between WLI and LCI.

**Figure 2:**
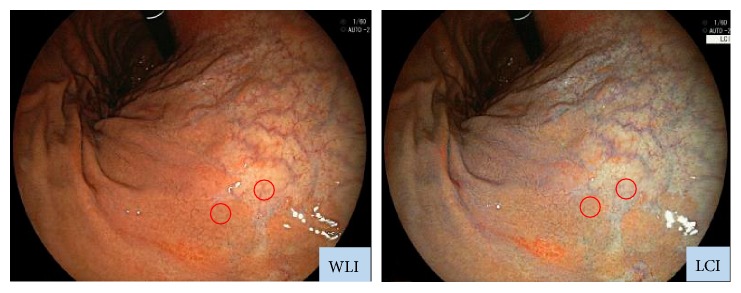
Typical images used for measurement of color differences at the atrophic border. Each sample area at the atrophic border was continuously imaged by WLI and LCI with the same composition. Then, the colors of the atrophic and nonatrophic mucosae were measured.

WLI and LCI were captured with the same light composition. In each image, sample areas of gastric regions containing the atrophic border (circles in Figure 2) were determined using the following procedure: (1) set a sample area of the atrophic mucosa by WLI image, (2) set another sample area in the nonatrophic mucosa adjacent to the first sample area, and (3) set the area corresponding to 2 sample areas which were determined by WLI as a sample area of LCI image. In each patient, we set 3 pairs of sample areas and calculated the color difference between atrophic and nonatrophic mucosal areas (e.g., color difference at the atrophic border) with each image of WLI and LCI. The endoscopic images were stored in the Joint Photographic Experts Group format, and each sample area was provided with a circle being inscribed to a square from 26 × 26 to 28 × 28 pixels.

**Figure 3:**
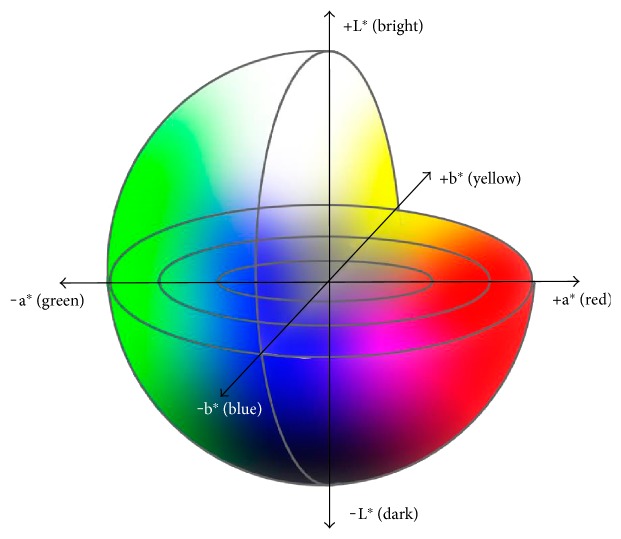
The color space according to the International Commission on Illumination 1976 definition (L^∗^a^∗^b^∗^). This color space is designed to approximate human perceptions. The Euclidean distance between two points is proportional to the difference in perception of the two corresponding colors.

Color differences (Δ*E*) were calculated using the International Commission on Illumination (CIE) 1976 (L^∗^, a^∗^, b^∗^) color space [15]. The CIE 1976 (L^∗^a^∗^b^∗^) color space is a 3-dimensional model composed of a black-white axis (L^∗^), a red-green axis (a^∗^), and a yellow-blue axis (b^∗^) (Figure 3). L^∗^ defines brightness, a^∗^ defines the red-green component, and b^∗^ defines the yellow-blue component. This color space is designed to approximate human perceptions, and the Euclidean distance between two points is proportional to the difference in perception of the corresponding colors. In other words, perceived color differences can be assessed in terms of Δ*E* values calculated based on the CIE 1976 (L^∗^, a^∗^, b^∗^) color space, which were previously shown to correspond to color differences in endoscopic images [16, 17].

Δ*E* was calculated via the following procedure: (1) The L^∗^a^∗^b^∗^ values were calculated from the red-green-blue values for each pixel in each of the atrophic and three nonatrophic sample areas in each image [18]. (2) The mean L^∗^, a^∗^, and b^∗^ were used as the L^∗^a^∗^b^∗^ values representative of the atrophic and nonatrophic mucosae in each image. (3) For each image, Δ*E* (e.g., the color difference at the atrophic border) was calculated using the following formula: {(ΔL^∗^)^2^ + (Δa^∗^)^2^ + (Δb^∗^)^2^}^1/2^, where ΔL^∗^, Δa^∗^, and Δb^∗^ are, respectively, the differences in the L^∗^, a^∗^, and b^∗^ values between the atrophic and nonatrophic mucosae. Pixels affected by halation were excluded.

### 2.4. Endpoints

The primary endpoint was a comparison of Δ*E* between WLI and LCI images collected in all patients (overall cohort). The secondary endpoint was a comparison of Δ*E* between WLI and LCI images in patients stratified according to *H. pylori* infection status (e.g., *H. pylori* infected and *H. pylori* eradicated). In addition, the visibilities of the atrophic border on LCI were assessed at points where the borders were difficult to determine on WLI. These borders were identified as the points showing Δ*E* < 5 on WLI, based on the CIE 1976 (L^∗^, a^∗^, b^∗^) color space.

### 2.5. Statistical Analysis

The Wilcoxon signed-rank test was used to compare Δ*E* values between LCI and WLI. A *P* value of less than 0.05 was considered to indicate statistical significance. Statistical analyses were performed using *R*, version 3.1.1.

## 3. Results

This study enrolled 30 patients (15 males, 15 females; mean age, 65.5 ± 13.5 years). According to the Kimura-Takemoto classification, mucosal atrophy was mild (C1, C2) in 4 patients, moderate (C3, O1) in 17, and severe (O2) in 9. Regarding *H. pylori* infection status, 9 patients had active infection, whereas *H. pylori* had been eradicated in 21 patients. The period following eradication was 6.13 ± 6.7 years (range 0–21 years) on average (it is unknown about 5 cases because they were eradicated at other hospitals).

**Figure 4:**
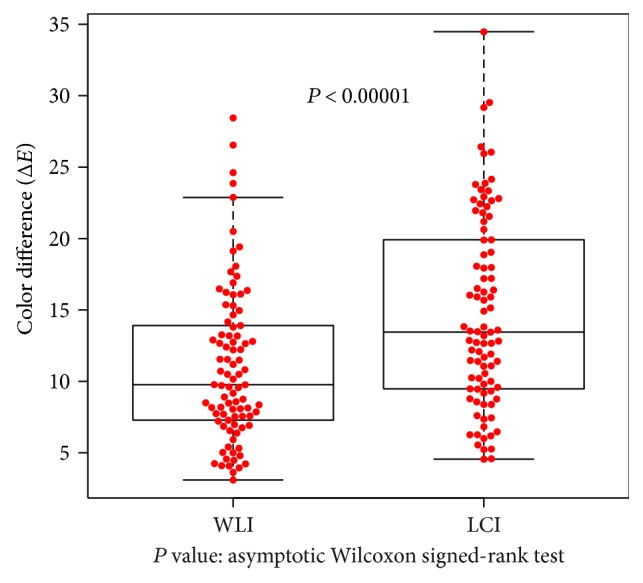
Color differences at the atrophic border examined by WLI and LCI in the overall cohort. Differences were computed between sample areas of the atrophic and nonatrophic mucosae. In 30 patients who provided consent, color differences expressed as Δ*E* and evaluated at 90 points were compared between WLI and LCI. Δ*E* values were significantly higher for LCI (*P* < 0.00001 by asymptotic Wilcoxon signed-rank test).

The color differences at the atrophic border in the overall cohort (30 patients; 90 points evaluated) make up the primary endpoint of this study; Δ*E* was 11.06 ± 5.44 on WLI and 14.79 ± 6.68 on LCI, which was significantly higher (*P* < 0.00001; Figure 4).

**Figure 5:**
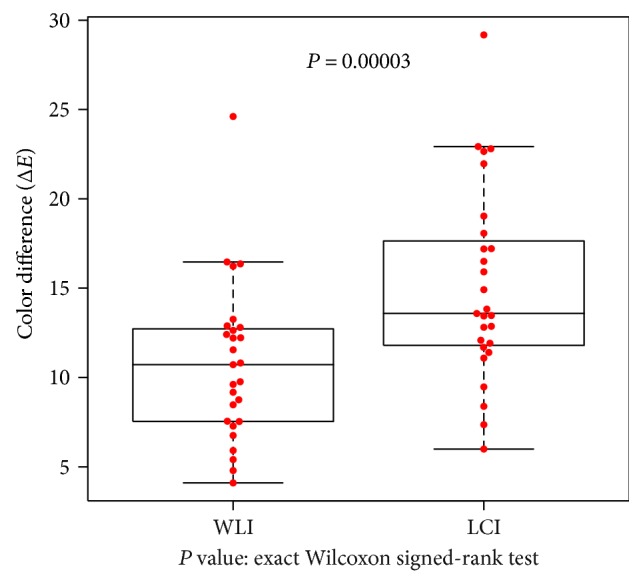
Color differences at the atrophic border examined using WLI and LCI in patients with active *H. pylori* infection. Differences were computed between sample areas of the atrophic and nonatrophic mucosae. In 9 patients with active *H. pylori* infection, color differences expressed as Δ*E* and evaluated at 27 points were compared between WLI and LCI. Δ*E* values were significantly higher for LCI (*P* = 0.00003 by exact Wilcoxon signed-rank test).

**Figure 6:**
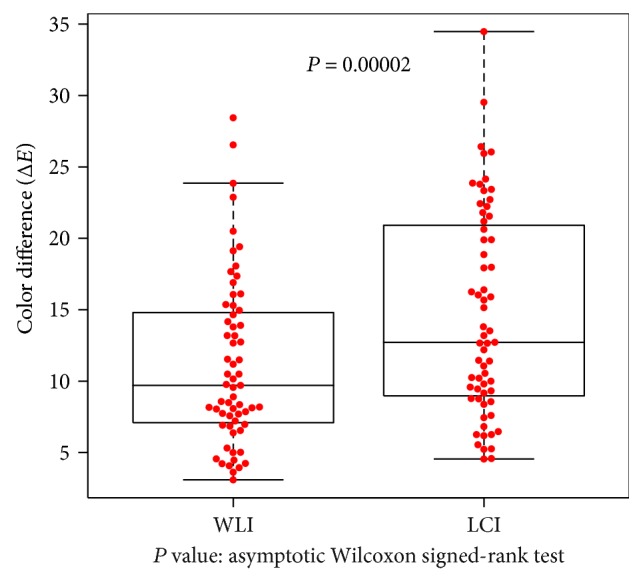
Color differences at the atrophic border examined with WLI and LCI in patients with eradicated *H. pylori* infection. Differences were computed between sample areas of the atrophic and nonatrophic mucosae. In 21 patients with a history of *H. pylori* eradication, color differences expressed as Δ*E* and evaluated at 63 points were compared between WLI and LCI. Δ*E* values were significantly higher for LCI (*P* = 0.00002 on asymptotic Wilcoxon signed-rank test).

The color differences at the atrophic border in the *H. pylori*-infected group (9 patients, 27 points evaluated) represent secondary endpoints of this study, Δ*E* was 10.8 ± 4.41 on WLI and 15.1 ± 5.4 on LCI, which was significantly higher (*P* = 0.00003; Figure 5). In the *H. pylori*-eradicated group (21 patients, 63 points evaluated), Δ*E* was 11.2 ± 5.58 on WLI and 14.7 ± 7.19 on LCI, which was significantly higher (*P* = 0.0002; Figure 6).

**Figure 7:**
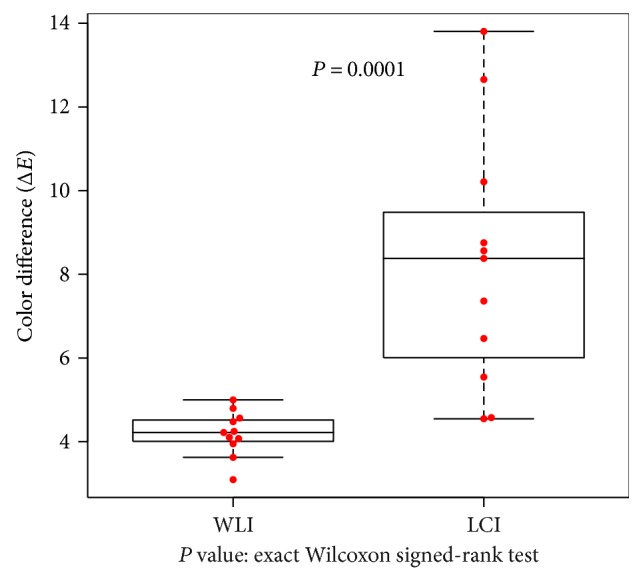
Visibility of the atrophic border examined with WLI and LCI in areas with very similar colors on WLI. Visibility was evaluated as color differences computed between sample areas of the atrophic and nonatrophic mucosae. Areas with similar colors on WLI were identified as those areas showing Δ*E* < 5 on WLI, based on the International Commission on Illumination 1976 (L^∗^, a^∗^, b^∗^) color space. In such areas, it is difficult to distinguish the atrophic border on WLI. A total of 11 points (evaluated in 7 patients) met this condition, and color differences expressed as Δ*E* were compared between WLI and LCI. Δ*E* values were significantly higher for LCI (*P* = 0.0001 on exact Wilcoxon signed-rank test).

We identified 11 points (in 7 patients) where the atrophic border was difficult to distinguish (Δ*E* < 5) on WLI. As for the other assessments, we found that, although Δ*E* was 4.2 ± 0.53 on WLI, the visibility of the border was significantly improved on LCI, which showed Δ*E* = 8.3 ± 3.05 (*P* = 0.0001; Figure 7).

## 4. Discussion

In this study, we demonstrated that the border between atrophic and nonatrophic mucosae in atrophic gastritis can be easily and clearly visualized using the LCI system. To our knowledge, this was the first study in which the LCI-based diagnosis of *H. pylori*-related gastritis was objectively assessed using quantified color values.

Prolonged infection with *H. pylori* causes atrophy of the gastric mucosa. From the histological point of view, mucosal atrophy is characterized by reduced density of gastric glands forming the mucosa, which results in shortening mucosal thickness [2]. Meanwhile, the findings of endoscopic mucosal atrophy include loss of mucosal folds, changes in mucosal colors, and appearance of vascular patterns through the transparent mucosa [11]. The line between mucosal areas with different colors is typically considered the mucosal atrophic border. LCI emphasizes differences in colors similar to those displayed by the mucosa on endoscopy, which is expected to enhance the detection of the atrophic border. The progression of atrophy is typically assessed according to the Kimura-Takemoto classification [11]. Because the grade of endoscopic atrophy correlates with that of histologic atrophy [19], our findings that LCI improves the visibility of the border of endoscopic mucosal atrophy suggest that LCI allows accurate determination of the status of gastritis.

**Figure 8:**
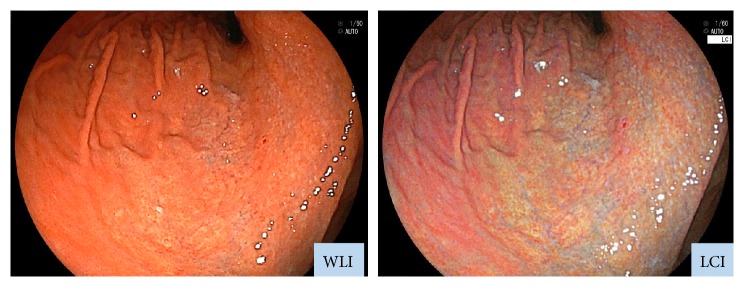
Typical images of atrophic gastritis in a patient with active *H. pylori* infection. (left) WLI and (right) LCI.

Moreover, our study demonstrated that LCI improved the visibility of the atrophic border regardless of *H. pylori* infection status. Diffuse redness is a typical finding of active *H. pylori* infection and indicates gastric mucosal inflammation accompanied by congestion or dilatation of capillary vessels in the superficial layer of the mucosa [3]. In the *H. pylori*-infected group, diffuse redness was observed in nonatrophic areas. Because LCI emphasizes redness (i.e., reddish areas become redder), the color difference between atrophic and nonatrophic areas presumably increases (Figure 8).

**Figure 9:**
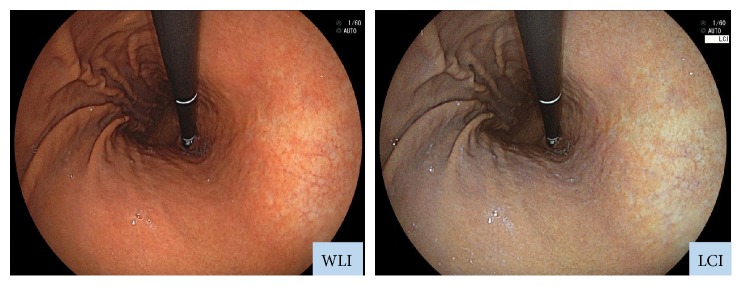
Typical images of atrophic gastritis in a patient with previous history of *H. pylori* eradication. (left) WLI and (right) LCI.

In this study, the *H. pylori*-eradicated group also showed significant differences in Δ*E* between WLI and LCI. Specifically, on LCI images of *H. pylori*-eradicated patients, nonatrophic mucosa appeared bluish (Figure 9). In our previous study, neutrophil infiltration was almost absent histologically immediately after eradication, and monocyte infiltration gradually resolved over 4 to 5 years. Additionally, long-term follow-up also revealed resolution of atrophy [20]. After *H. pylori* eradication, endoscopic findings reveal resolution of both inflammation and diffuse redness [21]. However, it was shown that atrophic mucosa that has changed in color may not be entirely healed; additionally, the atrophy-specific color often persists even after healing [20, 22]. By emphasizing these color differences, LCI seems to permit a higher visibility for such findings.

In our sample, we found that 11 points determined have low visibility on WLI, which led to difficulty in identifying the atrophic border; of these, 9 points were found in the *H. pylori*-eradicated group. We divided *H. pylori*-eradicated cases into two groups: One was eradicated within less than 5 years, and the other was eradicated more than 5 years ago. We investigated the visibility of atrophic border. However, in this study, we could not see a statistical significant difference on *ΔE* score of WCI. In general, after eradication, mucosal atrophy may resolve with the recovery of the function of gastric glands, which occurs over a long period of time [20, 23]. Since eradication therapy has been widely adopted, opportunities to observe gastric mucosa after eradication therapy are expected to increase in the future. In recent years, the features and other aspects characterizing the development of gastric cancer after *H. pylori* eradication have become the focus of intense research [23]. Meanwhile, the association between the incidence of gastric cancer after *H. pylori* eradication and the severity or resolution of atrophic gastritis has also been reported [23]. Moreover, because the typical endoscopic findings of gastric cancer lesions occurring after eradication consist of smaller size, flatter appearance, and less demarcated borders compared to those of ordinary gastric cancer [24–27], it is expected that gastric cancer after eradication will be difficult to detect. In EGD, we macroscopically identify lesions based on size, as well as based on the texture and color of the mucosal surface. The present study demonstrated that, under various gastric conditions, LCI emphasized subtle color differences and allowed to distinguish atrophic gastritis lesions.

Although conventional image-enhanced endoscopies such as narrow band imaging or blue laser imaging have been proven useful for qualitative diagnosis in combination with magnifying endoscopy [28, 29], the background images appear dark, which makes it difficult to observe the entire stomach. For this reason, such modalities are not suitable for screening for early gastric cancer [30]. On the other hand, LCI provides bright images that allow clear visualization of both the lesion and the background. LCI was previously reported to be useful for identifying local lesions including early gastric cancer and colon laterally spreading tumor [12, 13]. Taken together, the findings of the present study and those of the previous investigations suggest that LCI may be adopted as a technique to evaluate gastritis status and EGD screening for medical examination.

There are some limitations in this study. First, the present study is that the sample size was small. But this study sufficiently proved a statistical significant difference and showed its availability, so we think that it is a valuable pilot study. Second, we were unable to find histological support for the color differences highlighted on LCI. Future studies are expected awaited to investigate the histological significance of endoscopic findings enabled by LCI.

## 5. Conclusions

Our study found significant evidence that LCI allows clear endoscopic visualization of the atrophic border under various conditions of gastritis, regardless of the presence or absence of *H. pylori* infection or duration after eradication. Comparison with histology is necessary to validate these findings.
